# Paediatric palliative care following hospital discharge: Prevalence and factors associated with non-continuity of palliative care for children with cancer in Busoga sub-region-eastern Uganda; A mixed methods study

**DOI:** 10.1371/journal.pgph.0004210

**Published:** 2026-01-30

**Authors:** Miriam Ajambo, Savio Mwaka, Joseph Gavin Nyanzi, Damalie Nalwanga, Joyce Balagadde, Joseph Rujumba

**Affiliations:** 1 Division of Palliative Care, Department of Clinical Services, Directorate of Curative Services, Ministry of Health, Kampala, Uganda; 2 Department of Paediatrics and Child Health, Makerere School of Medicine, Makerere College of Health Sciences, Makerere University, Kampala, Uganda; 3 Non-Communicable Diseases Department, Infectious Disease Research Collaboration, Kampala, Uganda; 4 Wentz Medical Centre, Kampala Uganda; 5 Department of Paediatric Oncology, Uganda Cancer Institute, Kampala, Uganda; Yale University School of Medicine, UNITED STATES OF AMERICA

## Abstract

Palliative care (PC) is crucial for children with cancer to alleviate suffering and enhance quality of life. However, continuity of pediatric palliative care (PPC) can be disrupted by factors such as lack of knowledge, stigma, bureaucratic hurdles, inadequate referral systems, and staffing shortages. There is limited data on the prevalence and factors associated with non-continuity of PPC in Uganda. This study explores the prevalence and factors contributing to non-continuity of PC among children with cancer in Uganda with Busoga Region in Eastern Uganda as a case study.This cross-sectional mixed-methods study was conducted at three facilities; two specialized tertiary facilities managing pediatric cancer and one Hospice Centre. Data were extracted from online databases for 307 children treated from 2019 to 2023, of whom 80 were alive during the study. A semi structured questionnaire was administered to caregivers of 77 children while nine key informant interviews were done with health workers from the three study sites. Descriptive statistics summarized data as proportions or percentages, and bivariate analysis used crude odds ratios to identify significant associations. Key informant interviews were transcribed and analysed thematically using the socio-ecological model. The prevalence of non-continuity of PC was 96.1% (95% CI: 88.4-98.0). All children who did not continue with PPC had received no referral to their nearest PPC provider. Barriers identified included: individual-level gaps in caregiver knowledge; relationship-level issues such as inappropriate cultural beliefs; health system-level challenges like limited human resources, inadequate training and funding, poor coordination and referral pathways, and service access issues; and policy-level concerns, including the lack of a national palliative care policy. The high prevalence of non-continuity of PC for children with cancer in Busoga highlights significant deficiencies in integrating palliative care into pediatric oncology services in Uganda. Addressing these challenges requires urgent government action to enhance palliative care funding and resources.

## Introduction

Palliative care (PC) employs a holistic approach to improve the quality of life for patients suffering from serious illnesses like cancer, as well as their families, by addressing the psychosocial, spiritual, and physical challenges associated with the illness. Suffering is prevented or relieved through early diagnosis and appropriate management of pain and other distressing symptoms [1]. The World Health Organization (WHO) describes paediatric palliative care (PPC) as the total care of a child’s body, mind, and spirit [2]. PPC includes pain management, counselling, specialized care, and psychosocial and spiritual support [3].

Cancer is an increasing public health burden in Sub-Saharan Africa [3]. According to the WHO Global Initiative on Childhood Cancers 2018, approximately 400,000 children are diagnosed with cancer annually, with 80% of these cases occurring in low- and middle-income countries (LMICs). Official records from Uganda Cancer Institute show that an estimated 7000 children develop cancer every year of which 700 end up at the UCI. A study by Mutyaba and colleagues puts the incidence of paediatric cancer at 139 per one million children in Uganda. Using the census figures for Busoga by UBOS puts the incidence at 315 children with cancer [4–6].

Following diagnosis, all these children require PC, making integration of PPC into standard paediatric cancer care critically important [4,7]. Early initiation of PC for children with cancer results in improved symptom burden, pain control, and quality of life with decreased intensive procedures [8,9,10].

However, one major challenge in LMICs is the difficulty in accessing PC, which hampers the continuity of services upon patient discharge [8,11]. Palliative care is considered best practice and is increasingly implemented earlier in the trajectory of cancer [12]. To ensure adequate symptom management, prevent patient suffering, and establish a relationship of trust, continuity (under 14 days) of PPC after discharge from the hospital is key. There should not be a gap in continuity for more than 14 days following discharge [1]. Non-continuity of palliative care exceeding 14 days post-discharge risks inadequate symptom management and increased patient suffering in a vulnerable population. This threshold is defined to ensure seamless transitions from inpatient to ambulatory settings [5]. Access to PPC by children in LMICs is affected by low levels of awareness among health workers about palliative care and an inadequate number of palliative care service providers [13]. In Uganda, government support for PC has steadily increased over the years. However, the country still lacks a comprehensive national strategy and formal guidelines specifically for PPC. Currently, 13 PC centers provide critical support to pediatric patients.

These hospices, offering outpatient and community-based services, work alongside Uganda’s two main tertiary facilities—the Uganda Cancer Institute (UCI) and the Mulago National Referral Hospital Paediatric Haematology-Oncology Unit (MNRH-PHOU)—to ensure continuity of PPC for children with cancer following discharge. Staff at these centers are trained in PPC and can provide home visits for children following discharge, either upon notification from the tertiary hospitals or directly from caregivers. Some of the caregivers are instructed to go to the hospices for continuity of PC after discharge. Mulago National Referral Hospital (MNRH) has a palliative care team which is supported by the Makerere University Palliative Care Unit but these a trained primarily to offer adult PC. These provide all components of PC to inpatients. These include, pain management, psychosocial and spiritual support. Those at the end of life are given end of life care. All these facilities order oral liquid morphine from either National Medical Stores or Joint Medical Stores, free of charge since the morphine powder that is used by HAU to manufacture the liquid morphine is pre-paid by government.

Rays of Hope Hospice Jinja is a non-governmental organization founded in August 2005 to provide PC to patients with life-threatening/life-limiting illnesses, mainly cancer and HIV/AIDS. They treat and care for more than 430 patients in nine districts of Busoga Region across an area of 10,000 km2 and a population of 3.5 million people. RHHJ exists to provide holistic care to patients and their families. The organization’s main goal is to make the patients as comfortable as possible, adding life to their days. Services offered include; Advocacy, Campaigning/lobbying, Clinical/medical services, community PC services, counselling/bereavement, education, family support, Help/Information/advice, public awareness, research, respite or hospice services, training [6].

A study by Kagarmanova and colleagues on PC in Uganda indicates that up to 226 health facilities (both private and public) are accredited to order morphine for pain management but this is just one component of palliative care. Besides, the study did not highlight the capacity of these facilities to offer PPC [14].

Despite Uganda’s growing paediatric cancer burden, policy-relevant data on non-continuity of PPC post-discharge remain limited. Existing evidence focuses on morphine access among adult patients. This study seeks to ascertain the prevalence and factors associated with non-continuity of PPC post-discharge.

## Materials and methods

### Study design

This study employed a cross-sectional convergent parallel mixed methods design. It was conducted at two tertiary facilities providing PPC and one palliative care institution where downward referrals from the two facilities are directed. A search of the hospital electronic databases at the two tertiary facilities was conducted for children who presented from 2019 to 2023. Caregivers of these children and health workers providing PPC at the facilities were interviewed. A semi structured questionnaire was administered to caregivers of children from which quantitative data was obtained while for Key Informant Interviews (KIIs), an interview guide was used to collect qualitative data.

The quantitative and qualitative data were analysed separately and integrated during interpretation.

### Ethics

Ethical approval was obtained from the School of Medicine Research and Ethics Committee (SOMREC). SOMREC gave approval to access patients’ records, respective institutions gave administrative clearance to access patient records. IRB (SOMREC) gave waiver of consent from participants before accessing their data. Written informed consent was obtained from all participants (caregivers and key informants). Patients’ records were handled with confidentiality and privacy. The study did not involve minors, the caregivers who responded to the questionnaire were all adults.

### Study setting and participants

The population of interest included children with cancer from the Busoga sub-region in Eastern Uganda who were managed and discharged from UCI and MNRH-PHOU from 2019 to 2023 (whether or not in remission) and health workers providing PPC at the three study sites. The study was carried out at UCI, MNRH-PHOU and RHHJ.

The estimated number of children in this region (from UBOS estimates) is 2,392,450, representing 10.3% of the population of all children in Uganda, translating to approximately 332 cases of children with cancer based on the study by Mutyaba and colleagues [4,15].

### Inclusion criteria

- Caregivers of children who were discharged from either of the two tertiary facilities during the study period (whether or not in remission) and were documented residents of the Busoga sub-region.
- Palliative care service providers at any of the three facilities who provided informed consent to participate in the study.

### Exclusion criteria

- Caregivers of children who passed away after discharge or without telephone contact information or those that could not be reached.
- Palliative care providers not involved in PPC or had worked at the three facilities for less than two years

### Sampling strategies

#### Caregivers.

Contact lists from UCI and MNRH-PHOU identified caregivers of children from Busoga Sub-region treated for cancer (January 2019–December 2023). Records were searched consecutively, and caregivers were contacted by phone to verify the child’s survival status (via empathetic inquiry: “How is your child doing?”).

For caregivers of children who had passed on, a comforting message was given and the call terminated. Caregivers of children who were alive were asked if the child continued to receive PC and from where. They were then invited to UCI/MNRH-PHOU (within 30 km of Kampala) or RHHJ (Busoga). Unreachable contacts (after two calls within 72 hours) and those who declined participation were excluded. Sampling continued until 77 caregivers were recruited.

#### Key informants.

Nine providers were purposively selected from RHHJ, MNRH-CTU, and UCI (three per site: nurse, doctor, psychosocial worker with ≥2 years of experience) based on expertise and availability. Consenting participants were scheduled for KIIs.

### Data collection procedures

Patient records were accessed from 11/1/2024. Patient recruitment for interviews and questionnaire survey started on 12/1/2024 until 24/1/2024.

Quantitative Data Collection; Initial screening was done through a phone call, to the caregivers, to identify those with children still alive and these were enrolled into the study.

A semi-structured electronic questionnaire was administered using Kobo Collect by the PI with support from a research assistant in instances where the respondent did not understand English, a translated version of the Questionnaire was administered and then manually entered into the Kobo collect. The Questionnaire explored the patient demographics, patient level, health system and policy factors associated with non-continuity of PPC in Busoga sub-region.

The questionnaire was adapted from prior work by Levine and colleagues and made to align with the conceptual framework and deployed electronically into Kobo collect [16]. Prior to field testing, dummy data was entered to ensure correct functionality Field testing was done on five children attending Mulago Paediatric Haematology Oncology Unit but from regions other than Busoga. Data was reviewed from which the questionnaire was further refined and a final version was deployed into Kobo collect.

Qualitative Data Collection**;** Interview guides were used for KII. The interviews were conducted by the PI with support from research assistants with experience in conducting qualitative interviews in palliative care contexts. The Key informants were interviewed from their respective places of work, on appointment. Each interview took about 30–45 minutes. The KII guide explored health system, patient level and policy related factors. These interviews were recorded verbatim with the consent of the participants.

The interview guide was generated by the Principal Investigator (PI) for the purpose of the study and reviewed for consistency with the objectives by subject matter experts in the area of Paediatric Oncology and Qualitative methods. Feedback from the subject matter experts was incorporated into the final version

Data Quality Control**;** Daily data reviews were conducted by the study team. These involved checking submitted quantitative data for completeness and adherence to predefined data quality checks embedded in the electronic data collection system. Where inconsistencies were identified, corrective actions included providing immediate feedback to data collectors. To prevent interviewer bias and ensure alignment between transcripts and recordings, a different team was responsible for transcribing the interviews than the one that conducted them.

### Data analysis

Quantitative data analysis was performed using STATA 15. Descriptive statistics for categorical data were summarized as proportions or percentages and presented using tables. Crude odds ratios were used to establish bivariate relationships. Non-continuity of palliative care was operationalized as a binary outcome variable. Children were coded as having non-continuity of care if no palliative care follow-up contact was reported after discharge from the tertiary facility. The prevalence of non-continuity was calculated as a proportion, with the numerator being the number of children who did not continue PC and the denominator being the total number of children in the study [17].

The recordings were transcribed verbatim by research assistants, after which the PI verified the transcripts for accuracy. The PI then familiarized themselves with the data through multiple readings, noting recurring patterns. An inductive coding process was employed, beginning with initial codes generated by the PI, which were reviewed and refined by an independent qualitative researcher. Following this feedback, the PI conducted further iterative readings and coding to develop a finalized coding frame, which was then applied by the PI to the remaining data. This single-coder approach with expert peer review ensured rigor and reflexivity throughout the thematic analysis. Verbatim quotations reflecting key informant views on factors associated with the non-continuity of PPC were identified and used in presentation of study findings. These barriers and facilitators were analysed using the socio-ecological model (SEM), exploring factors at the individual, relationship, health system, and policy levels [18]. The socio-ecological model (SEM) was used to analyse the qualitative data because continuity of paediatric palliative care is influenced by multiple interacting factors at individual, interpersonal, health system, and societal levels. The SEM provides a structured framework for organizing barriers and facilitators across these levels, allowing for a comprehensive and context-sensitive understanding of factors shaping continuity of PPC in the Busoga Sub-Region.

## Results

A total of 307 children were discharged from the two tertiary facilities between 2019 and 2023. Of these, 153 (49.8%) had died, and 74 (24.1%) were unreachable. Among the 80 eligible participants, 77 consented and were enrolled.

**Fig 1:**
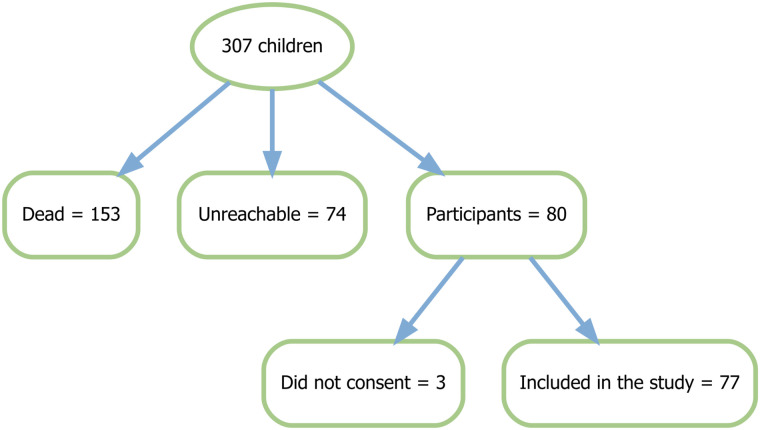
Participant enrolment.

**Fig 1** is a STROBE diagram for patient enrolment.

### Prevalence of non-continuity of PC

Of the 77 caregivers enrolled, 74 (96.1%; CI: 88.4 -98.8) reported that their children did not continue to receive PC services.

### Relationship between patient characteristics and non-continuity of PPC

Seventy-four out of 77(96.1%) children did not continue to receive palliative care after discharge, 36/74(48.6%) of these were not in school. However, all three who continued to receive PC were also not in school. Of those that did not continue, 33/74(44.6%) had liquid tumours and of these, 20/33(60.6%) had non-Hodgkin’s lymphoma, predominantly Burkitts Lymphoma. None of the characteristics showed a statistically significant relationship. Table 1 shows the relationship between patient characteristics and non-continuity.

**Table 1:** Relationship between patient characteristics and non-continuity.

Variable	Continued, N = 3(3.9%), n(column%)	Didn’t continue, N = 74, n(column%)	COR(95%CI)	p value
**Age**				0.800
0-4 years	0(0.0)	21(28.4)	1.000	
5-9 years	1(33.3)	21(28.4)	0.429(0.024,7.632)	0.564
10-14 years	1(33.3)	23(31.1)	0.391(0.022,6.949)	0.523
15-17 years	1(33.3)	9(12.2)	1.000	
**Gender**				0.801
Female	1(33.3)	30(40.5)	1.000	
Male	2(66.7)	44(62.9)	1.364(0.118,15.722)	0.804
**Diagnosis**				
**Solid tumours**				
Bone tumour	0(0.0)	1(1.4)		
Soft tissue sarcoma	0(0.0)	9(12.2)		
Brain tumour	0(0.0)	5(6.8)		
Wilm’s tumour	0(0.0)	14(18.9)		
**Liquid tumours**				
Leukaemia	0(0.0)	13(17.6)		
Non-Hodgkin’s	0(0.0)	20(26.0)		
Others	3(100)	12(16.2)		
**Child’s class**				
Not in school	3(100)	36(48.6)		
Pre-primary	0(0.0)	7(9.5)		
Lower primary	0(0.0)	18(24.3)		
Upper primary	0(0.0)	13(17.6)		
**Duration of symptoms**				0.362
< 3 months	0(0.0)	29(39.2)	1.000	
3- < 6 months	2(66.7)	13(17.6)	3.077(0.253,37.483)	0.378
6 < 1 year	0(0.0)	12(16.2)	1.000	
1 year +	1(33.3)	20(27.0)	1.000	

### Relationship between caregiver characteristics and non-continuity of PPC

Seventy out of 77 (90.9%) caregivers in the study were biological parents. Among these, 95.7% did not continue with PC. All children whose caregivers were not their biological parents did not continue with PC. None of the caregiver characteristics showed a statistically significant relationship. Table 2 presents the caregiver characteristics and their relationship with non-continuity of PC

**Table 2:** Relationship between caregiver characteristics and non-continuity.

Variable	continued, N = 3(3.9%), n(column%)	Didn’t continue, N = 74, n(column%)	N = 77(100%)	COR(95%CI)	p
**Caretaker relationship**					
Parent	3(100.0)	67(90.5)	70(90.9)		
Grandfather	0(0.0)	4(5.5)	4(5.2)		
Other relatives	0(0.0)	3(4.1)	2(2.6)		
**Caretaker marital status**					
Married/cohabiting	3(100)	63(85.1)	66(85.7)		
Separated/divorced	0(0.0)	5(6.8)	5(6.5)		
Single	0(0.0)	4(5.4)	4(5.2)		
Widowed	0(0.0)	2(2.7)	2(2.6)		
**Education level of household head**					0.792
No formal education	0(0.0)	7(9.5)	7(9.1)	1.000	
Primary education	2(66.7)	32(43.2)	34(44.2)	1.375(0.117,16.111)	0.800
Secondary education	1(33.3)	22(29.7)	23(29.9)	1.000	
Tertiary education	0(0)	13(17.6)	13(16.9)	1.000	
**Caregiver occupation**					0.414
Business person	1(33.3)	8(10.8)	9(11.7)	1.000	
Civil servant	0(0.0)	6(8.1)	6(7.8)	1.000	
Others	0(0.0)	11(14.9)	11(14.3)	1.000	
Peasant	2(66.7)	49(66.2)	51(66.2)	0.383(0.026,4.034)	0.328
**Setting**					
Rural	3(100)	42(56.8)	45(58.4)		
Urban	0(0.0)	32(43.2)	32(41.6)		

### Relationship between health system factors and non-continuity of PPC

Those who did not receive PC information from Health workers had higher odds COR; 22.667(95% CI 1.785,287.820), p = 0.016 of non-continuity of PC. All three that continued had been given PC information by the healthcare workers.

All the 74 that did not continue PC were not given referral information for continuity of PC at discharge but all the three that continued received it. Two were referred to RHHJ and one, to Jinja RRH.

### Qualitative analysis results from key informant interviews

Nine KI were selected from the three study sites. Table 3 presents the demographic characteristics of the key informants.

**Table 3:** Demographic characteristics of the Key informants.

	MNRH-PHOU N = 3(33.3%)	UCI N = 3(33.3%)	RHHJ N = 3(33.3%)	N = 9(100%))
**Sex**				
Male	1	1	0	2(22.2)
Female	2	2	3	7(77.8)
**Years in service**				
< 5	1	1	0	2(22.2)
5-9	2	1	2	5(55.6)
10-14	0	1	1	2(22.2)
**Designation**				
Nursing officer	1	1	0	2(22.2)
Social worker	0	1	1	2(22.2)
PC clinical Officer	0	0	2	2(22.2)
Medical officer	1	1	0	2(22.2)
Paediatric haematologist oncologist	1	0	0	1(11.1)
**Palliative care training**				
Diploma	2	1	3	6(66.7)
certificate	1	1	0	2(22.2)
No training	0	1	0	1(11.1)

### Descriptive category: Informants’ understanding of palliative care

As part of the contextual findings, key informants were asked to describe their understanding of palliative care. Overall, most informants demonstrated a good understanding of palliative care as a holistic and integrated approach to improving the quality of life of patients with life-threatening or chronic illnesses and their families. Palliative care was described as routine care that should be provided from the time of diagnosis through to end of life, with attention to symptom management, psychosocial support, cultural factors, and family involvement.

Several informants emphasized pain and symptom control as core components of palliative care, alongside longitudinal follow-up:

**“***It is an approach which gives quality of care to the patient with life-threatening illnesses; patients and their families, identifying their symptoms, and managing the pain is very important, then following them up through diagnosis to the end-of-life.”* ***(KII 1, MNRH)***

*Others highlighted the comprehensive nature of palliative care beyond physical symptoms:*

*“Palliative care basically focuses on comprehensive support in terms of pain management and other psychological aspects given to people with life-threatening illnesses such as cancer, people with HIV, diabetes, sometimes with trauma after accidents.”* ***(KII 4, UCI)***

However, a minority of informants demonstrated a limited understanding of palliative care, viewing it primarily as care provided at the end of life rather than as an approach integrated throughout the disease trajectory:

*“Ideally, I would take palliation as the care at the end of life for someone who we believe we are not going to do much in terms of curing them. We don’t start it at the start of treatment.”* ***(KII 6, UCI)***

This variation in understanding provided important context for interpreting subsequent findings related to continuity of pediatric palliative care.

## Theme 1: Barriers and facilitators to continuity of pediatric palliative care for children with cancer in the Busoga Sub-Region

This theme captures factors that either hindered or supported the continuity of PPC for children with cancer. The identified barriers and facilitators were analysed using the SEM adapted from Pierce and Kealey (2023), encompassing individual, relationship, health system, and societal levels.

### Sub-theme 1: Individual-level barriers to continuity of PPC

#### Knowledge gaps among caregivers.

A knowledge gap among caregivers emerged as a key barrier to continuity of PPC, as limited understanding of palliative care affected caregivers’ acceptance of services, adherence to follow-up schedules, and sustained engagement with care after referral or discharge. Key informants explained that caregivers who lacked literacy or awareness were more likely to disengage from care, resulting in missed appointments and treatment abandonment. Informants noted that caregivers cannot engage with or sustain palliative care services if they are unaware of their purpose or benefits.

*“Sometimes it is illiteracy, most patients’ attendants may not know that palliative care can help somebody to have a quality life or live long.”* ***(KII 2, MNRH)***

Limited understanding of palliative care was also linked to poor acceptance and treatment abandonment:

*“Knowledge gap and acceptance! If people lack the knowledge and understanding of what palliative care is, they don’t accept… When linking up these patients, they get lost along the way—others refuse and abandon treatment, probably due to fear.”* ***(KII 3, MNRH)***

#### Childhood developmental factors.

Challenges in assessing and interpreting pain in children were reported to affect continuity of PPC by limiting timely symptom reassessment and adjustment of care plans during follow-up. Difficulty in accurately assessing pain, particularly after discharge or between visits, was described as contributing to delays in care escalation and inconsistent symptom control, which undermined ongoing engagement with palliative care services.

*“It is very hard to understand how children experience pain… Sometimes the child is crying and you think it’s pain yet the child needs to shower.”* ***(KII 4, UCI)***

### Sub-theme 2: Socio-cultural barriers influencing continuity of PPC

#### Social challenges.

Social disruptions following a childhood cancer diagnosis such as parental separation or divorce were reported to hinder continuity of care. Informants emphasized that fragmented family structures compromise care-seeking, follow-up, and financial support.

*“When a child is diagnosed with cancer, the man will divorce the mother… When the family is shattered it affects the continuity.”* ***(KII 1, MNRH)***

#### Cultural beliefs and explanatory models.

Cultural beliefs attributing cancer to witchcraft or moral transgressions were reported to divert caregivers away from biomedical care, leading to discontinuity of PPC.

*“Most of them think it is witchcraft… when they are discharged from here, they go and look for other ways witch doctors.”* ***(KII 3, MNRH)***

### Sub-theme 3: Health system related barriers to continuity of PPC

#### Limited human resources for pediatric palliative care.

Shortages of trained PPC providers and the absence of designated PPC focal persons were major barriers. Informants described overwhelming workloads and reliance on borrowed staff from adult services, resulting in delayed and inconsistent care.

*“We need a focal person… but we still don’t have one for pediatrics.”* ***(KII 4, UCI)***

Even where trained personnel existed, competing clinical duties limited their ability to conduct follow-up:

*“We are overwhelmed… Others we forget to follow up because we are overwhelmed with work.”* ***(KII 3, MNRH)***

#### Knowledge gaps among health workers.

**Insufficient training among health workers limited the integration of PPC into routine care, despite some individual competence**

*“There is a knowledge gap among health workers… We need to bridge that gap so that all health workers are able to consider palliative care as part of routine care.”* ***(KII 2, MNRH)***

#### Limited prioritization of palliative care.

The lack of dedicated PPC units, committees, and coordination structures led to fragmented services and weak follow-up mechanisms.

*“For children it’s not a well-organized service… we still have scattered elements of palliative care.” (* ***KII* 4, UCI)**

#### Limited access to services.

Frequent transfers of trained staff and limited availability of essential medicines such as morphine at lower-level facilities further disrupted continuity of care.

“Morphine is cost-free but sometimes in those facilities, the medicines aren’t there.” (KII 4, UCI)

#### Stigmatization of palliative care.

Some healthcare professionals perceive PPC as less valuable or “useless,” leading to demoralization and the reassignment of PPC providers to other departments.

“*We are being fought when you want to do it [palliative care], our fellow medical workers say ‘why are you doing palliative care? That is useless!’ They even remove one from palliative care units and take them to surgery, medical because they think you are not performing! They don’t see it as something that is important, thus hindering continuity of the service”* ***KII 1, MNRH.***

#### Lack of child-friendly services.

A few key informants noted the unavailability of child-friendly services. Facilities often lack specialized clinics or spaces tailored to children’s needs, resulting in fear and reluctance among children to attend follow-up appointments.

*“We don’t have a specialized clinic for children! We only handle the children in the same clinic with the adults-there is no space so we end up sharing the same space -when you look around there are no child friendly services so when a child comes here it knows its injections yet we don’t inject here! But because they have been in those procedures, they come here very frightened and it’s really hard to relieve their fears, this hinders continuity of PC among the children”* ***KII 09, RHHJ***.

#### Challenges in accessing referred facility for PPC.

Key informants identified difficulties that patients and caregivers face in locating and accessing the specific palliative care facilities to which they are referred after discharge. This lack of clear guidance and support in the referral process often results in patients becoming lost“to follow-up, disrupting the continuity of care**.**

*“People get lost- they can be given a referral from the cancer institute to hospice Jinja and the care taker fails to locate us. A patient may be discharged from the cancer institute and not sent to any specific palliative care facility they continue coming to UCI for review but when they aren’t aware of any palliative care Centre where they can access care within that period thus they don’t continue with PC”* ***KII 08 RHHJ.***

#### Poor service proximity resulting into high transport costs.

Most of the key informants stated that, children stay far from the facility and its costly to transport them from home to the facility and this leads to non-continuity of PC.

*“We are also challenged in a way that it’s not easy for us to have children in one place -having children care in a particular place would be very good but because of the diverse distance it’s hard to collect one from Namayingo, two from Mayuge and bring them together so we are challenged! The fuel, you have to transport them and this gets costly for the organization”* ***KII 08 RHHJ.***

#### Communication challenges.

Most of the key informants noted how difficult it is for children to continue PC when there is a communication barrier between that PC providers and the caregivers. Some of the caregivers do not have phones and yet for others, language barrier also poses a great challenge. As patients don’t get to understand the health worker’s instructions in regards to continuity of palliative care.

“*Most of them don’t even have phones and telephone numbers. There is one I really hustled, the telephone number in the file was for the neighbor who had even shifted to another place”* ***KII 1, MNRH.***

#### Inadequate funding/budgetary allocation for palliative care.

Most of the key informants alluded to inadequate funds or government budgetary allocation towards PC as a key hindrance to the continuity of PPC. On some occasions, they noted to have used their own funds to follow-up patients in their homes or by phone calls.

*“When the patients go home it’s really hard. There is no provision for following up or visiting patients at home. Sometimes, we really suffer as palliative care people; when there is a patient who needs home visiting, I go for a home visit, I pull money from my own pocket. Sometimes I follow them up using my money…”* ***KII 1, MNRH***

### Sub-theme 4: Health system–related facilitators to continuity of PPC

In contrast to the barriers described above, key informants also identified several health system related facilitators that supported continuity of pediatric palliative care, particularly through collaboration, flexible service delivery models, and active follow-up mechanisms.

#### Collaborative Partnerships with Community-Based Organizations.

Strong partnerships with hospices and non-governmental organizations were reported to play a critical role in facilitating continuity of care beyond the hospital setting. These collaborations supported home-based care, follow-up, psychosocial support, and access to essential medications.

*“We try our level best through collaborations, phone calls. When we get children from those particular areas where we have collaborators, especially hospices to do home visits, we coordinate… We facilitate provision of painkiller particularly morphine and get a lot of help from Hospice Uganda.”* ***(KII 2, MNRH)***

#### Use of mixed models of care delivery.

Informants described the use of mixed models of care including hospital-based, hostel-based, and community-based approaches to address diverse clinical and psychosocial needs. This flexibility was particularly important for children at end of life or those with complex family and bereavement challenges.

*“We offer hospital-based, hostel-based with collaboration with Bless the Child, community-based with collaboration with Rays of Hope and other palliative care providers… mainly for patients at end-of-life, those with complex psychosocial and bereavement issues.”* ***(KII 3, MNRH)***

#### Active follow-up through phone communication.

Despite resource constraints, phone communication was commonly used to maintain contact with patients and caregivers after discharge. Health workers reported using phone calls to support medication adherence, coordinate drug delivery, and provide ongoing psychosocial support, thereby enhancing continuity of care.

*“If they need drugs, we collaborate with the taxi guys and put them on the taxi and the drugs reach them or we write a prescription and then follow up with phone calls.”* ***(KII 3, MNRH)***

#### Mapping and liaising with local palliative care services.

Efforts to identify and link patients to palliative care services closer to their homes were described as an important facilitator of continuity. Mapping local services enabled timely referrals and reduced the burden of travel for families.

*“We try to map out palliative care services within the locality of those children… Busoga region has organized palliative services! Jinja, there is Rays of Hope, Arua has Joy Hospice, Hoima, Mbarara, Hospice Hoima.”* ***(KII 4, UCI)***

#### Institutional support and training.

Institutional commitment, including staff training in palliative care and the provision of free morphine, was reported to strengthen PPC delivery and referral processes. Informants noted that shared knowledge among staff and access to essential medicines facilitated more coordinated and responsive care.

*“The hospital is very supportive in the sense that we have been trained and we all have an idea on palliative care services and this makes our work easy. Second, there is free morphine for pain relief. Third, since we use a mixed approach, these other community-based [services] help us continue care.”* ***(KII 2, MNRH)***

The quantitative and qualitative findings converged conceptually. While quantitative analyses showed no statistically significant associations between patient or caregiver characteristics and non-continuity of PPC, health system factors particularly lack of information about PC and referral were strongly associated with non-continuity. The qualitative findings provided explanatory depth by demonstrating how gaps in knowledge, referral processes and system capacity undermine continuity of care after discharge

## Discussion

This study shows a high prevalence of non-continuity of PC in Busoga sub-region with 96% of children not continuing to receive PC services after discharge. From the caregiver perspective, non-continuity was largely driven by health system related factors, particularly the failure to provide adequate information about PC and clear referral pathways at health facilities.

The prevalence of non-continuity observed in this study is higher than that reported by Rehner and colleagues in Mecklenburg Western Pomerania, where 79% of children did not continue palliative care, and exceeds findings from another Ugandan study indicating that only about 10% of individuals in need of palliative care receive such services [5,14]. These comparisons underscore substantial gaps in continuity of PPC within the Busoga sub-region and highlight the critical role of health system processes in sustaining care beyond hospital discharge. In a scoping review on barriers to the provision and utilization of palliative care in Africa, Agom and colleagues identified health education and timely referral as key factors influencing utilization PC. Although this review was not specific to PPC, the findings align with our study, where inadequate information and unclear referral pathways were major contributors to non-continuity of care from the caregivers’ perspective [19]

Key informants attribute caregivers’ reluctance to continue PC to cultural beliefs such as attributing cancer to witchcraft or supernatural punishment for wrongdoing. Such beliefs hinder the continuity of PC, leading to unmanaged pain and other challenges. This finding is consistent with Hawley’s findings in the study on barriers to access to palliative care which reported reluctance to accept PC referrals due to cultural beliefs in Denmark. Similarly, a study in Ethiopia found that patients’ preference for conventional medicine was a barrier to PC services [20,21].Cultural beliefs significantly influence caregivers’ acceptance and continuation of paediatric palliative care, acting as a critical barrier to effective care delivery.

The study revealed that a cancer diagnosis often leads to social challenges such as parental separation or divorce, which disrupts the necessary support system. This family breakdown can leave the child vulnerable to non-continuity of PC, as single parents may struggle with transport and emotional support. Mader and colleagues found that parents under 45 years who were unemployed prior to the child’s cancer diagnosis had a higher risk of separation or divorce. Haimi and Lerner also highlighted that divorce can exacerbate children’s mental health issues and increase non-continuity of PC [22,23]. Given that cancer diagnosis is associated with most of these mental challenges, parent separation without full family support, interruptions in care-seeking become inevitable due to issues like difficulty in raising finances.

The study identified several healthcare system barriers, including limited human resources, inadequate training, and insufficient funding for PC. The shortage of skilled personnel in PPC and the lack of training and expertise could be attributed to inadequate funding and low interest among medical personnel in PPC due to its emotional challenges. Limited human resources affect service continuity, as there is inadequate follow-up and a risk of missing referral information. Similar challenges were noted by several researchers including Mosoiu and colleagues in Romania and Abate in Ethiopia, who cited health care provider turnover, shortage of healthcare workers, and lack of government support. Insufficient training of health professionals in LMICs has also been reported as a major obstacle [20,24]. Health system constraints, particularly limited human resources, inadequate training, and insufficient funding, critically undermine the delivery and continuity of PPC.

Structural issues such as the absence of a national PC policy lead to inconsistent service delivery, inadequate resource allocation and limited advocacy and research. A national PC policy is crucial for guiding the management of PC, ensuring strategic prioritization and mobilizing resources. The WHO’s 2016 public health approach to PC emphasized the need for such policies. Policies like Kenya’s PC policy, launched in 2021, provide better guidance for resource allocation and service delivery [25–27].

Caregivers’ understanding of PC significantly facilitated its continuity. All caregivers of children who continued to receive PC had some level of understanding of PC, while those who did not continue had no information about it. This finding aligns with findings by Hudson and colleagues which emphasized that informed caregivers contribute to effective care [28]. A study by Wu and colleagues also revealed that caregivers are essential in PC provision and must possess appropriate knowledge, a compassionate and positive attitude and take care of their emotional, mental, social, and physical well-being which facilitate continuity [29]. Caregivers who understand what palliative care involves are more likely to seek out palliative care providers for the patient even after discharge, being aware that such services can enhance the patient’s quality of life.

The findings underscore the critical role of collaborative partnerships and innovative care models in enhancing the continuity and quality of PPC in resource-limited settings. Strong collaborations between tertiary hospitals and community-based organizations, including hospices and NGOs, extend the reach of PPC beyond hospital walls, enabling home-based care, follow-up and psychosocial support. A study by Carroll and colleagues also highlights the importance of this integration of care across the sites of care that range from hospital to home [30]. This partnership model not only facilitates access to essential medications such as morphine but also leverages local resources to provide comprehensive care tailored to patients’ needs. As one key informant noted, coordination with hospices and community actors is vital for delivering pain relief and maintaining contact with families, highlighting the importance of integrated networks in overcoming geographic and resource barriers. Another study by Kamal and colleagues emphasizes the importance of Community-based PC programs as an important component of a public health approach to palliative care and thus, the importance of linkages between hospitals and community based organizations cannot be under estimated [31]. Hospices and community-based PC providers play a crucial role in supporting patients after discharge, as they are situated closer to the patients’ homes and communities. This proximity allows for more personalized, accessible and continuous care, which tertiary healthcare facilities highly value. These PC providers not only manage symptoms and provide emotional support but also coordinate with hospitals to ensure seamless transitions and avoid unnecessary readmissions. By being embedded within the community, they are better positioned to address patients’ holistic needs, including social, psychological, and spiritual aspects of care. Many of these providers also engage in education and advocacy to improve the overall quality of EOL.

Mixed care delivery models, integrating hospital-based, hostel-based and community-based services, enhance the responsiveness of PPC to children’s complex needs, particularly at end-of-life or in psychosocially challenging situations. Evidence from New Zealand shows that effective liaison with community-based services, combined with comprehensive assessment and management of pain, improves the well-being of children and their families [32]. These findings highlight the importance of coordinated referral systems and community partnerships to ensure sustained, patient-centered PPC [33].

Key informants reported that follow-up phone calls are routinely used to support treatment adherence, medication access and psychosocial care despite limited resources. Evidence shows that post-discharge telephone follow-up is a cost-effective intervention for improving care transitions [34]. In this study, phone-based follow-up emerged as a pragmatic strategy for maintaining engagement with caregivers, bridging geographical barriers and reducing loss to follow-up, thereby supporting continuity of care after discharge [35]. Although telephone follow-up has limitations such as lack of direct phone access for some caregivers and reliance on alternative contacts the benefits outweigh these challenges.

The availability of free oral morphine supports collaborative and community-based PC efforts by enabling effective pain and symptom control. The World Health Organization emphasizes access to essential medicines, particularly oral morphine, as fundamental to quality palliative care and a core component of a comprehensive approach to strengthening palliative care services [36].

Collectively, these findings highlight that a multifaceted approach, anchored in collaboration, flexible service delivery, proactive follow-up, strategic mapping, and institutional commitment, is vital for overcoming barriers to PPC continuity. Scaling up such models could improve access, quality, and sustainability of pediatric palliative care in similar low-resource contexts

**Study Strengths**; Study represents the first investigation into non-continuity of PPC in Uganda and is among the few studies on PPC in East Africa.

**Study Limitations**; The high mortality rate limited the population from which a sample could be drawn, making it impossible to perform a multivariable statistical analysis. Additionally, recall bias may have affected the accuracy of information, as participants had to remember what they were told upon discharge. Selection bias was also a concern, as those who had died were excluded from the study. Furthermore, some confounders could not be measured or controlled due to the retrospective nature of the study. This study was not able to distinguish between children whose PC needs may have resolved following discharge and those who continued to require PC but did not receive services, potentially leading to an overestimation of non-continuity of PC.

## Conclusion

This study reveals a critical deficiency in the continuity of PC for children with cancer in the Busoga sub-region, with over 96% failing to receive care after hospital discharge. This gap leaves many children vulnerable to diminished quality of life. Inadequate health education and unclear referral pathways were associated with non-continuity of PPC.

From the KIIs, cultural misconceptions and family disruptions further impede caregivers’ ability to continue care, while health system limitations such as insufficient trained personnel, inadequate funding and poor coordination exacerbate service fragmentation. The absence of a national policy on PC compounds these challenges by limiting strategic planning and resource mobilization.

Nonetheless, the study identifies promising facilitators: caregiver awareness, strong partnerships between hospitals and community organizations, flexible care delivery models, proactive follow-up mechanisms, and institutional support through training and medication access. These elements demonstrate the potential to bridge existing gaps.

To improve PPC continuity, urgent multi-level interventions are necessary. These include formal integration of PPC into healthcare structures with dedicated teams, enhanced workforce capacity building, development of a national PC policy, and strengthened linkages between tertiary centers and community-based services. Empowering caregivers through education and fostering community collaborations will also be key to sustaining care. Implementing such comprehensive strategies is vital to ensuring that children with life-limiting illnesses receive consistent, compassionate, and accessible PC.

## Supporting information

S1 Text Questionnaire. The questionnaire collected data on demographics, patient-level factors, health facility factors, and policy-level factors influencing continuation or non-continuation of PPC. Sections include child’s sex, religion, cancer type, school status, caregiver relationship and demographics, household information, symptoms duration, awareness and knowledge of PC referral and enrollment details, current access to palliative care.
(DOCX)

S2 Text Key informant interview guide. This appendix contains the structured interview guide used to collect qualitative data from healthcare providers involved in PPCservices. It outlines the flow of the interview covering introduction, respondent particulars (initials, designation, workstation), duration of service, perceptions of PC, existence and functioning of PC committees, training in PC, models of care delivery, factors affecting PPC service provision, barriers to patient access following discharge, and recommendations for improving PPC continuity.
(DOCX)

S1 Table Codebook. The codebook outlines the thematic framework developed from qualitative analysis of key informant interviews regarding PPC services. It includes major themes, sub-themes, definitions, and exemplar quotes from transcripts illustrating each concept.
(DOCX)
