# Supplementary material for: Paediatric palliative care following hospital discharge: Prevalence and factors associated with non-continuity of palliative care for children with cancer in Busoga sub-region-eastern Uganda; A mixed methods study
Source: PLOS Glob Public Health. 2026 Jan 30;6(1):e0004210. doi: 10.1371/journal.pgph.0004210 (PMC12858009; doi:10.1371/journal.pgph.0004210)
Supplement: S1 Text — The questionnaire collected data on demographics, patient-level factors, health facility factors, and policy-level factors influencing continuation or non-continuation of PPC. Sections include child’s sex, religion, cancer type, school status, caregiver relationship and demographics, household information, symptoms duration, awareness and knowledge of PC referral and enrollment details, current access to palliative care. (DOCX) [file pgph.0004210.s001.docx]

Appendix III: Questionnaire

**Prevalence and factors associated with non-continuity of Palliative Care for children with cancer in Busoga sub-Region-Eastern Uganda.**

Interviewer ID:

Participant ID:

Interview Date:

Demographics and patient level factors

1. Sex of the child

a. Male

b. Female

2. Child’s religion

a. Catholic

b. Anglican

c. Muslim

d. SDA

e. Orthodox

f. Pentecostal

g. Other (specify)

3. Type of cancer

a. Leukaemia

b. Burkitts lymphoma

c. Non-Hodgkin’s Lymphoma

d. Bone tumour

e. Brain tumour

f. Spinal cord tumour

g. Retinoblastoma (eye cancer)

h. Hepatocellular carcinoma (liver cancer)

i. Soft tissue cancer

j. Other (specify)

4. Is the child in school?

a. Yes

b. No

5. If yes, what class?

a. Pre-primary

b. Lower primary(P.1-P.4)

c. Upper primary(P.5-P.7)

d. O’level

e. A’ level

6. Is the child currently studying in Busoga sub-region or elsewhere (Specify)?

a. Yes

b. No

7. What is your relationship with the child?

a. Father

b. Mother

c. Uncle

d. Aunt

e. Cousin

f. Sibling

g. Grand father

h. Grand mother

i. Not biologically related

8. Marital status of care giver:

a. Single

b. Married/cohabiting

c. Separated/Divorced

d. Widow/widower

9. Educational level of household head

a. No formal education

b. Primary education

c. Secondary education

d. University/Tertiary education

10. Number of people in the household.

………………………………………………………………………

11. Occupation of the care giver

a. Peasant

b. Civil servant

c. Business person

d. Other (specify)

40

12. How much is your household expenditure per month in Uganda Shillings

a. Less than 100,000

b. 100,000-300,000

c. 300,001-500,000

d. More than 500,000

13. Is the respondents home in an Urban or rural setting?

a. Urban

b. Rural

14. How long did the child have symptoms before he was diagnosed with cancer?

a. < 3 months

b. 3-<6 months

c. 6< 1 year

d. 1 year +

15. Have you ever heard about palliative care?

a. Yes

b. No

16. If yes, what do you know about palliative/supportive care?

……………………………………………………………………………………………

17. When your child was finally diagnosed with cancer, did the health worker tell you about

palliative care/ supportive care?

a. Yes

b. No

18. If yes, what did the health worker tell you about palliative/supportive care?

a. Support for people with incurable illness

b. Support for people going to die

c. A service that focuses on pain relief

d. PC comprises of; Counselling/social support, pain relief, spiritual nourishment,

legal support and end of life care, for individuals with life limiting illness.

41

19. On discharge, did you receive information on where you should go, to continue

palliative care services?

a. Yes

b. No

20. If yes, which facility were you referred to for the continuation of Palliative/supportive

care?

a. At Uganda Cancer Institute

b. At JRRH

c. At Rays of Hope Hospice Jinja

d. At the nearest General Hospital

e. Other facility

21. What did you anticipate when the health worker referred you for palliative care/

supportive care?

……………………………………………………………….

22. Did you eventually enrol for the Palliative care/supportive care services?

a. Yes

b. No

23. If yes, at which facility are you receiving continuation of Palliative care/supportive care?

a. At Uganda Cancer Institute

b. At JRRH

c. At Rays of Hope Hospice Jinja

d. At the nearest General Hospital

e. Other facility

24. If no, why didn’t you enrol for the palliative care/ supportive care services?

………………………………………………………………………..

If no skip to 25

25. If yes, how long did it take you to enrol for these services?

a. < 2weeks

b. 2weeks-<3months

c. 3-<6 months

d. 6< 1 year

e. 1 year +

26. Why did you take this long to access these services?

a. I did not think they are useful

b. The facility I was referred to was far away and I couldn’t afford transport fair

c. The facility I was referred to did not have the services

d. I was so depressed and needed some time to get back to normal

e. I took long to trace the facility I was referred to

f. I opted for services by a traditional healer first

27. Are you currently accessing PPC services?

a. Yes

b. No

28. If yes, where?

a. RHHJ

b. JRRH

c. UCI

d. MNRH-PHEOU

e. Others (specify)

29. If no, why

f. I did not think they are useful

g. The facility I was referred to was far away and I couldn’t afford transport fair

h. The facility I was referred to did not have the services

i. I was so depressed and needed some time to get back to normal

j. I took long to trace the facility I was referred to

k. I opted for services by a traditional healer first

Health Facility factors

30. Which services in particular are you accessing at the facility?

a. Pain management

b. Counselling

c. Spiritual support

d. Holistic care

31. For how long have you been accessing these services?

a. < 3 months

b. 3-<6 months

c. 6< 1 year

d. 1 year +

32. Since you started accessing these services, how useful have you found these services?

a. Not useful at all

b. Somewhat useful

c. Useful

d. Very useful

33. What services in particular have you found useful?

a. Pain therapy

b. Counselling

c. Spiritual support

d. All

34. What benefit have you achieved from each of these Palliative/supportive care services

accessed

a. Access to pain medications

b. Psycho-social support

c. Spiritual nourishment

d. All the above

For each of these palliative/supportive care services, how do you think they can be improved

35. Pain therapy?

a. Train health workers on pain management

b. Avail pain medications at health facilities

c. Other. Specify

36. Counselling and spiritual support

a. More counsellors and spiritual leaders should involved

b. Facilitate counsellors and spiritual leaders to conduct community visits due to

long distances to facilities

c. Other. Specify.

Policy level factors

37. If you were to meet the health Minister, what three barriers would you request her to

prioritize to better continuity of paediatric palliative/supportive care services in Busoga

sub-region?

a. Reduce on stock outs of pain medications like oral morphine.

b. Increasing access to palliative care at nearest facilities

c. Recruiting more health workers to provide palliative care services

d. Need to train more health workers in palliative care

e. Collaborate with more NGOs to provide palliative care services

f. More sensitization and advocacy for palliative care to increase public awareness

g. More male involvement in palliative care services

h. Improve capacity of health facilities to detect cancer early

i. Support Palliative Care policy development

38. Would you recommend a colleague with a similar problem to enrol for

palliative/supportive care services?

a. Yes

# b. No Appendix III: **Questionnaire**

Prevalence and factors associated with non-continuity of Palliative Care for children with cancer in Busoga sub-Region-Eastern Uganda**.**

Interviewer ID:

Participant ID:

Interview Date:

**Demographics and patient level factors**

1. Sex of the child
2. Male
3. Female
4. Child’s religion
5. Catholic
6. Anglican
7. Muslim
8. SDA
9. Orthodox
10. Pentecostal
11. Other (specify)
12. Type of cancer
13. Leukaemia
14. Burkitts lymphoma
15. Non-Hodgkin’s Lymphoma
16. Bone tumour
17. Brain tumour
18. Spinal cord tumour
19. Retinoblastoma (eye cancer)
20. Hepatocellular carcinoma (liver cancer)
21. Soft tissue cancer
22. Other (specify)
23. Is the child in school?
24. Yes
25. No
26. If yes, what class?
27. Pre-primary
28. Lower primary(P.1-P.4)
29. Upper primary(P.5-P.7)
30. O’level
31. A’ level
32. Is the child currently studying in Busoga sub-region or elsewhere (Specify)?
33. Yes
34. No
35. What is your relationship with the child?
36. Father
37. Mother
38. Uncle
39. Aunt
40. Cousin
41. Sibling
42. Grand father
43. Grand mother
44. Not biologically related
45. Marital status of care giver:
    1. Single
    2. Married/cohabiting
    3. Separated/Divorced
    4. Widow/widower
46. Educational level of household head
    1. No formal education
    2. Primary education
    3. Secondary education
    4. University/Tertiary education
47. Number of people in the household.

………………………………………………………………………

1. Occupation of the care giver
2. Peasant
3. Civil servant
4. Business person
5. Other (specify)
6. How much is your household expenditure per month in Uganda Shillings
   1. Less than 100,000
   2. 100,000-300,000
   3. 300,001-500,000
   4. More than 500,000
7. Is the respondents home in an Urban or rural setting?
   1. Urban
   2. Rural
8. How long did the child have symptoms before he was diagnosed with cancer?
   1. < 3 months
   2. 3-<6 months
   3. 6< 1 year
   4. 1 year +
9. Have you ever heard about palliative care?
   1. Yes
   2. No
10. If yes, what do you know about palliative/supportive care?

……………………………………………………………………………………………

1. When your child was finally diagnosed with cancer, did the health worker tell you about palliative care/ supportive care?
   1. Yes
   2. No
2. If yes, what did the health worker tell you about palliative/supportive care?
3. Support for people with incurable illness
4. Support for people going to die
5. A service that focuses on pain relief
6. PC comprises of; Counselling/social support, pain relief, spiritual nourishment, legal support and end of life care, for individuals with life limiting illness.
7. On discharge, did you receive information on where you should go, to continue palliative care services?
8. Yes
9. No
10. If yes, which facility were you referred to for the continuation of Palliative/supportive care?
    1. At Uganda Cancer Institute
    2. At JRRH
    3. At Rays of Hope Hospice Jinja
    4. At the nearest General Hospital
    5. Other facility
11. What did you anticipate when the health worker referred you for palliative care/ supportive care?

……………………………………………………………….

1. Did you eventually enrol for the Palliative care/supportive care services?
   1. Yes
   2. No
2. If yes, at which facility are you receiving continuation of Palliative care/supportive care?
   1. At Uganda Cancer Institute
   2. At JRRH
   3. At Rays of Hope Hospice Jinja
   4. At the nearest General Hospital
   5. Other facility
3. If no, why didn’t you enrol for the palliative care/ supportive care services?

**………………………………………………………………………..**

***If no skip to 25***

1. If yes, how long did it take you to enrol for these services?
   1. < 2weeks
   2. 2weeks-<3months
   3. 3-<6 months
   4. 6< 1 year
   5. 1 year +
2. Why did you take this long to access these services?
   1. I did not think they are useful
   2. The facility I was referred to was far away and I couldn’t afford transport fair
   3. The facility I was referred to did not have the services
   4. I was so depressed and needed some time to get back to normal
   5. I took long to trace the facility I was referred to
   6. I opted for services by a traditional healer first
3. Are you currently accessing PPC services?
4. Yes
5. No
6. If yes, where?
7. RHHJ
8. JRRH
9. UCI
10. MNRH-PHEOU
11. Others (specify)
12. If no, why
13. I did not think they are useful
14. The facility I was referred to was far away and I couldn’t afford transport fair
15. The facility I was referred to did not have the services
16. I was so depressed and needed some time to get back to normal
17. I took long to trace the facility I was referred to
18. I opted for services by a traditional healer first

**Health Facility factors**

1. Which services in particular are you accessing at the facility?
   1. Pain management
   2. Counselling
   3. Spiritual support
   4. Holistic care
2. For how long have you been accessing these services?
   1. < 3 months
   2. 3-<6 months
   3. 6< 1 year
   4. 1 year +
3. Since you started accessing these services, how useful have you found these services?
   1. Not useful at all
   2. Somewhat useful
   3. Useful
   4. Very useful
4. What services in particular have you found useful?
   1. Pain therapy
   2. Counselling
   3. Spiritual support
   4. All
5. What benefit have you achieved from each of these Palliative/supportive care services accessed
   1. Access to pain medications
   2. Psycho-social support
   3. Spiritual nourishment
   4. All the above

For each of these palliative/supportive care services, how do you think they can be improved

1. Pain therapy?
   1. Train health workers on pain management
   2. Avail pain medications at health facilities
   3. Other. Specify
2. Counselling and spiritual support
   1. More counsellors and spiritual leaders should involved
   2. Facilitate counsellors and spiritual leaders to conduct community visits due to long distances to facilities
   3. Other. Specify.

**Policy level factors**

1. If you were to meet the health Minister, what three barriers would you request her to prioritize to better continuity of paediatric palliative/supportive care services in Busoga sub-region?
   1. Reduce on stock outs of pain medications like oral morphine.
   2. Increasing access to palliative care at nearest facilities
   3. Recruiting more health workers to provide palliative care services
   4. Need to train more health workers in palliative care
   5. Collaborate with more NGOs to provide palliative care services
   6. More sensitization and advocacy for palliative care to increase public awareness
   7. More male involvement in palliative care services
   8. Improve capacity of health facilities to detect cancer early
   9. Support Palliative Care policy development
2. Would you recommend a colleague with a similar problem to enrol for palliative/supportive care services?
   1. Yes
   2. No
