# Supplementary material for: Paediatric palliative care following hospital discharge: Prevalence and factors associated with non-continuity of palliative care for children with cancer in Busoga sub-region-eastern Uganda; A mixed methods study
Source: PLOS Glob Public Health. 2026 Jan 30;6(1):e0004210. doi: 10.1371/journal.pgph.0004210 (PMC12858009; doi:10.1371/journal.pgph.0004210)
Supplement: S2 Text — This appendix contains the structured interview guide used to collect qualitative data from healthcare providers involved in PPCservices. It outlines the flow of the interview covering introduction, respondent particulars (initials, designation, workstation), duration of service, perceptions of PC, existence and functioning of PC committees, training in PC, models of care delivery, factors affecting PPC service provision, barriers to patient access following discharge, and recommendations for improving PPC continuity. (DOCX) [file pgph.0004210.s002.docx]

Appendix V: **KEY INFORMANT INTERVIEW GUIDE**

Title of the survey; **Prevalence and factors associated with non-continuity of Palliative Care for children with cancer in Busoga sub-Region-Eastern Uganda**

Flow of the Interview;

1. Introduce yourself: Hello…I am .…

Introduce the project: Prevalence and factors associated with non-continuity of Palliative Care for children with cancer in Busoga sub-Region-Eastern Uganda.

1. Date of the Interview _____/_______/______

1. Respondent’s Particulars

| Initials of respondent __________________ | Title/ Designation  ____________________ | Work station   - RHHJ - UCI - MNRH-PHOU |
| --- | --- | --- |

1. How long have you worked at this Institution?
2. What does Palliative Care mean to you?
3. Do we have a functional PC committee/ team at the facility?
4. If yes, how often does the committee meet? (ask to have a look at the minutes)
5. What is the composition of the committee in terms of designations?
6. Have you been trained in Palliative Care?
7. What model of Palliative Care delivery do you use? Facility or community-based? Explain
8. What factors do you consider to affect delivery or overseeing of PPC services at your facility?
9. What do you think are the factors that affect patients’ access to PPC after discharge from MNRH-PHOU and/or UCI?
10. What are some of the issues you would want the Ministry of Health PC division to address in order to improve continuity of Paediatric Palliative Care in Busoga sub-region.
