# Supplementary material for: Paediatric palliative care following hospital discharge: Prevalence and factors associated with non-continuity of palliative care for children with cancer in Busoga sub-region-eastern Uganda; A mixed methods study
Source: PLOS Glob Public Health. 2026 Jan 30;6(1):e0004210. doi: 10.1371/journal.pgph.0004210 (PMC12858009; doi:10.1371/journal.pgph.0004210)
Supplement: S1 Table — The codebook outlines the thematic framework developed from qualitative analysis of key informant interviews regarding PPC services. It includes major themes, sub-themes, definitions, and exemplar quotes from transcripts illustrating each concept. (DOCX) [file pgph.0004210.s003.docx]

CODEBOOK

| Themes | sub-themes | Definition | Examples from transcripts |
| --- | --- | --- | --- |
| Participants' Understanding of PC | Good understanding | participants' ability to define palliative care conventionally | “It is an approach which gives quality of care to the patient with life-threatening illnesses; patients and their families, identifying their symptoms, and managing the pain is very important, then following them up through diagnosis to the end-of-life” **-KII 1, nursing officer** |
|  |  |  | “Basically, to me, palliative care is an art of routine medical care that should be given to all patients with either chronic conditions or life-threatening conditions! It entails looking at those social aspects of life, the cultural stressors and the support that you really give to the patient and the family from the time of diagnosis till death of the patient”-**KII 2, medical doctor** |
|  |  |  | “Actually, me as a person whenever am reviewing my patients, I look beyond the diagnosis, beyond the chemotherapy. I look at their psychological needs, religious, cultural things -I do more of talking with the patients and the family and from that you identify a lot of things that might have interfered with the treatment or something that’s stressing the patient or the family”-**KII 2, medical doctor** |
|  |  |  | “Palliative care basically focuses on comprehensive support in terms of pain management and other psychological aspects given to people with life-threatening illnesses such as cancer, people with HIV, diabetes, sometimes with trauma after accidents”**-KII 4, medical social worker** |
|  | Poor Understanding | participants' inability to define palliative care conventionally | “Ideally, I would take palliation as the care at the end of treatment for someone who we believe we are not going to do much in terms of curing them. We don’t start it at the start of treatment”**-KII 6, medical officer** |
| A) Barriers |  |  |  |
| a. Individual level | Chilhood developmental aspects | Child's developmental aspects that hinder continuity of PC | “It is very hard to understand how children experience pain! Sometimes the child is crying and you think it’s pain yet the child needs to shower! So those elements make it very hard to know the level of pain”-KII 4, medical social worker |
|  | Social challenges | Issues in the child's social cycles, that can hinder continuity of PC | “Sometimes, most patients have social problems- when a child is diagnosed with cancer, the man will divorce the mother saying they don’t give birth to cancer patients. When the family is shattered it affects the continuity” -KII 1, nursing officer |
| b. Relationship factors | Knowledge gap on palliative care among care givers | care givers limited/ inadequate understanding of palliative care | “Sometimes it is illiteracy, most patients’ attendants may not know that palliative care can help somebody to have a quality life or live long” **KII 2, Medical officer** |
|  |  |  | “Knowledge gap and acceptance! If people lack the knowledge and understanding of what palliative care is, they don’t accept! Many people see palliative care as end-of-life coz that’s when we come in mainly! That’s when start seeking care… When linking up these patients, they get lost along the way- there are some you link up others refuse and abandon treatment, probably due to fear.”-**KII 3, haemato-oncologist.** |
|  | Inappropriate cultural beliefs | Beliefs in Busoga culture that make caregivers prefer other modalities, besides PC and thus hinder PC continuity | “Beliefs; some don’t believe in palliative care, so after you have given them advice, you link them up but when they go, they do something else. Most of them think it [cause of cancer] is witchcraft and when they are discharged from here, they go and look for other ways- they go to witch doctors. I got a family that had separated because the child had cancer and the mother was accusing the dad that this child got cancer because he cheated! During bereavement, it took us time for almost a year and a half to ensure that these people were back together and I was happy for the success”-**KII 3, haemato-oncologist.** |
|  | Unwillingness of caregivers to continue PC | Situations where caregivers hestate to continue palliative care | “In the process of supporting if the caretaker drops out, don’t expect this child to continue, the child will remain home much as the child would have wished to continue with treatment and therefore you can’t coerce them you handle them the way they are”. KI 007 |
| **c)** Health system related factors | Limited human resource for paediatric palliative care. | Inadequate number of health workers with paediatric pallitive care knowledge | “Actually, we are supposed to have a palliative care unit and we are supposed to report and have a palliative care person or nurse on every unit but things aren’t properly streamlined. We are supposed to work as a team but now I work alone! I report to myself, I do everything myself I make decisions myself so I get overwhelmed and this affects PC continuity”**KII 1, nursing officer** |
|  |  |  | “I am particularly not doing palliative care. I carry out other procedures like giving chemo! When I came here, the director of this unit told us we would be managing patients who are at end -of-life but for me I know I am supposed to manage patients from diagnosis, but I didn’t want to conflict with…”**-KII 1, nursing officer** |
|  |  |  | “We have one palliative care nurse but instead of doing palliative care work, they gave her clinical work! Initially we had started palliative care work in oncology with a book to follow up patients but we gave up. We are overwhelmed. Others we forget to follow up because we are over whelmed with work so the challenges are really many…”**-KII 3, haemato-oncologist** |
|  |  |  | “Clinical work takes over, and we do less of palliative care. Sometimes we follow up a few and or call in the Mulago palliative care team. It’s kind of not easy -for those with sickle cell and hematological illnesses, I know even managing pain is palliative care but introducing the aspect of palliative care, we are not doing it, we are overwhelmed”-**KII 3, paediatric haemato-oncologist** |
|  | Knowledge gap among health workers | Health worker's limited/ inadequate understanding of palliative care | “I feel there is knowledge gap among health workers! I told you I can do the palliative care assessment and but am not sure whether everyone else does it! We need to bridge that gap so that all health workers are able to consider palliative care as part of routine care. The other challenge would be staff! For you to be able to deliver good quality palliative care you need people who are dedicated to the service-palliative care alone, who will sit down with the patients and families at different phases of treatment.”-KII 2, medical doctor |
|  |  |  |  |
|  | Lack of Palliative care prioritization | A facility's inability to treat palliative care as a priority | “The cancer institute hasn’t generally gone far in terms of palliative care services because a facility like this is supposed to have a fully-fledged unit or department dedicated to handle the palliative care aspect but…”**-KII 4, medical social worker** |
|  |  |  |  |
|  |  |  | “But for children it’s not a well-organized service because we don’t have a committee to comprehensively look at palliative care aspects; we still have scattered elements of palliative care. Normally, when we have serious palliative care cases, we tend to rush and borrow support from other sections to come and support”**KII 4, medical social worker** |
|  |  |  |  |
|  |  |  | “In terms of human resource, we need a focal person, an expert to guide us but we still don’t have the one for pediatrics! That affects us and that’s why we tend to rush quickly to the adult section to borrow someone, so it delays the process of administering timely care”**-KII 4, medical social worker** |
|  |  |  |  |
|  | Limited acess of services | PPC services are not readily available when needed by patients | “Palliative care persons are stationed in the original district headquarters or in the original referral hospitals- those ones in HCIII or HCIV. You contact and they will tell you am no longer there we are not in existence. Morphine cost-free but sometimes in those facilities the medicines aren’t there. We will try and get 3 or 4 bottles of morphine put it on the bus”-**KII 4, medical social worker.** |
|  |  |  |  |
|  | Stigmatisation of Palliative | Some Health workers consider PC to be of no importance | “We are being fought when you want to do it [palliative care], our fellow medical workers say ‘why are you doing palliative care? That is useless!’ They even remove us from palliative care units and put you in surgery, medical because they think you are not performing! They don’t see it as something that is important, thus hindering continuity of the service”**-KII 1, nursing officer.** |
|  |  |  |  |
|  |  |  |  |
|  | Lack of child-friendly services | Limited facilities tailored to children's needs. | “We don’t have a specialized clinic for children! We only handle the children in the same clinic with the adults-there is no space so we end up sharing the same space -when you look around there are no child friendly services so when a child comes here it knows its injections yet we don’t inject here! But because they have been in those procedures, they come here very frightened and it’s really hard to relieve their fears”. **-KII 09 –RHHJ.** |
|  |  |  |  |
|  | Navigation challenges | How patients trace out the fcaility where they have been referred to obtain palliative care | “People get lost- they can be given a referral from the cancer institute to hospice Jinja and the care taker fails to locate us. A patient may be discharged from the cancer institute and not sent to any specific palliative care facility they continue coming to UCI for review but when they aren’t aware of any palliative care centre where they can access care within that period-KII 08-RHHJ. |
|  |  |  |  |
|  |  |  | “Uncertainty of how they are going to receive the treatment the other side! You find someone in Buyende and this person has never even reached Jinja! You are telling this person cross to UCI and access treatment! This hinders us because you are willing to support but this person says I have never reached Kampala even jinja the person has never reached there! So the child ends up being there, you can’t coerce a parent because they have a right as these are their children”-KII 08-RHHJ. |
|  |  |  |  |
|  | Poor service proximity resulting into high transport costs | Long distances to facilities where palliative care services are obtained by patients | “We are also challenged in a way that it’s not easy for us to have children in one place -having children care in a particular place would be very good but because of the diverse distance it’s hard to collect one from Namayingo, two from Mayuge and bring them together so we are challenged! The fuel, you have to transport them and this gets costly for the organization”-KII 08- RHHJ |
|  |  |  | “Yes! That’s the time when the guardian or the care taker can’t afford -some will tell you am willing but financially I am down-so we support the parent or guardian and in-most cases if we got places in Kawempe home care it will be a bonus because our costs reduce so that we can support other children” KII 07-RHHJ. |
|  |  |  |  |
|  | Communication challenges | Palliative care providers encounter dificulties in conveying pallitive care information to the participants | “Most of them don’t even have phones and telephone numbers. There is one I really hustled, the telephone number in the file was for the neighbor who had even shifted to another place”-KII 1, nursing officer |
|  |  |  |  |
|  |  |  | “Of course, linkage gaps is number one, then communication gaps, language barrier. Some patients lack autonomy; it is the parents who decide for them- even if they leave when we have talked to them, they go home and do what they [the parents] wish”. -KII 3, paediatric oncologist |
|  |  |  |  |
|  |  |  | “The transport challenge that I stated before on the side of patients, but also other things like patients’ acceptance! Some relatives would really look at it as a burden, also their understanding of what palliation is, we also have the language barrier issue-you explain to someone, they seem to have understood but when they go home, they do different things and since we don’t do much follow-up, we don’t know what’s happening” -KII 6, medical doctor. |
|  |  |  |  |
| Policy related barriers | Inadequate funding/ budgetary allocation for palliative care | Finances allocated to palliative care are not sufficient | “We don’t have enough finances to do the community- based visits for all our patients and we would like to improve on that especially children who are at end-of-life. The parents think that we tend to ignore them more when we send them home; that would be improved if we had more finances” KII 2, medical officer |
|  |  |  |  |
|  |  |  | “When the patients go home it’s really hard. There is no provision of following up or visiting patients at home. Sometimes, we really suffer us palliative care people; when there is a patient who needs home visiting, I go for a home-visit, I pull money from my own pocket. Sometimes I follow them up using my money…’-KII 1, nursing officer |
|  |  |  |  |
|  |  |  | “Because of resource constraints; the airtime for follow-ups, home visits etc., all these need money. We find it very difficult to do home visits. Home visits are very nice because through them we are able to map out the resources that are available within the community to be able to support patients”-KII 4, medical social worker |
|  |  |  |  |
| B) Facilitators | Care coordination and referral pathway-PC beyond and after discharge from the hospital | How the MNRH-PHOU and UCI link/refer patients to lower facilities or do follow-up of patients and send morphine | “We go through clinics put the medicine on the bus and then we tell them [patient/family] to get the medicine from such and such a clinic. If the patient dies, we do bereavement on phone with the family. As Pead oncology, they also follow up and call them up for their schedules of treatment but for us they don’t give us airtime for palliative care’’-KII, 1, nursing officer |
|  |  |  |  |
|  |  |  | “The community based is of course more challenging but we try our level best through collaborations, phone call. When we get children from those particular areas where we have got collaborators, especially hospices to do for us the home visits, we coordinate. For example, for patients in Eastern Uganda we coordinate Rays of Hope, Kitovu mobile in greater Masaka… We facilitate provision of painkiller particularly morphine and we get a lot of help from hospice Uganda Sometimes we may have to put the morphine on private means or a taxi and send it to the community when the patient is stuck there without morphine” -KII 2, medical doctor |
|  |  |  |  |
|  |  |  | The support we offer is linkage and also collaboration. Some of them come through Hospice e.g. Rays of hope in Busoga; so even at discharge, we link them back to the local hospice. If they need drugs, we collaborate with the taxi guys and put them on the taxi and the drugs reach them or we write a prescription and then follow up with phone calls” -KII 3, paediatric haemato-oncologist |
|  |  |  |  |
|  |  |  | “We offer hospital based, hostel based with collaboration with bless the child, community based with collaboration with Rays of hope and other palliative care providers in the community! Then also phone call follow- up, so I should say its mixed but it’s mainly for patients at end-of-life, those with complex psycho social and bereavement issues”-KII 3, paediatric haemato-oncologist |
|  |  |  |  |
|  |  |  | “We try to map out palliative care services which are within the locality of those children. We tend to liaise with hospices and send patients through their unit to handle the rest of the things like home visits. For instance, Busoga region, Busoga region has organized palliative services! Jinja, there is Rays of Hope, Arua has Joy Hospice, Hoima Mbarara, Hospice Hoima…”-KII 4, medical social worker |
|  |  |  |  |
|  |  |  | “A patient for total palliation who has challenges with transport, we try as much as possible to get transport services for this patient! Some of them need food… we don’t do all that by ourselves here but we try as much as possible to map up and liaise with the communities where they are coming from to ensure sustainability and care continuity”-KII 4, medical social worker |
|  |  |  |  |
|  |  |  | “The hospital is very supportive in the sense that we have been trained and we all have an idea on palliative care services and this makes our work easy. Second, there is free morphine for pain relief. Third, since we use a mixed approach, in delivering this, these other community-based palliative care services help a lot and this makes the referral system”-KII 4, medical social worker |
|  |  |  |  |
| Suggestions to improve continuity of paediatric palliative care | Palliative Care Financing | There is need for more funds committed to Palliative care by government | “If the ministry and the government could dedicate some funds to facilitate hospitals to be able to deliver, at least touch on the community aspect of palliative care -I know there is money that comes as part of primary health care but I don’t think palliative care is part of it. And also supporting community-based organizations; I know they are struggling yet they are doing a good job. if the government can find a way of getting involved with them and patch up some gaps, I think it would be good”-KII 2, Medical officer |
|  | Palliative Care Policy | The need for a statutory document to guide palliative care service provision in Uganda | “Uganda as a nation does not have a palliative care policy. We need to have a palliative care policy to guide service delivery. The government needs to carry out advocacy-to educate, sensitise people. The sensitization program-like the way they are doing on malaria, Covid…, HIV”-KII 4, medical social worker |
|  | Education and awareness | There is need to increase communityknowldge on cancer and palliative care. | “Educating the community about cancer -it’s the most difficult thing we are dealing with in this palliative care settings-giving the people info about cancer, how it can be treated and prevented and if possible the services should be brought closer to the people. People cant afford going to UCI for treatment so if its brought nearer, more children can access the needed care as the go through this” -KII 08 |
|  |  |  | “The civil society and the ministry should create awareness of these non-communicable diseases- they should make them know that when I take care of you, I won’t get cancer by washing or touching your wound. There are those who are abandoned in their homes that oh I will get the same condition”-KII 07 |
|  | Treatment proximity | Need to bring palliative care services closer to the people | “Making treatment and services accessible -bringing them nearer to the people and this means putting more money in the health- related sector so that the children can get the best health care because they are the future generation-one thing I know is that for the children once treated early they survive. Good enough most of them that we have been sending they have improved! We want to thank the government for providing the chemotherapy for the children -let them work on the investigations the diagnosis because they are still a big challenge if they are to be treated in time”- KII 08-RHHJ |
|  |  |  | “These people stay in the villages and they walk very many kilometers to find a health facility where they could have these investigations made so we wish the ministry of health could help setup some health facilities that have all the machinery that are needed to see that people can access care near - by -someone tells you I didn’t know where to go and even you can drive to a patient’s home and you wonder if you are still on track! You wonder if you who is in the vehicle you are complaining about the distance what about this person who has to walk and if they are lucky to have a motocycle or a bicycle to the facility that’s great”-KII 07-RHHJ |
|  | Support with logistics and supplies | support to palliative care institutions with all materials required for pallitaive care provision | “Supporting the hospices-if there is any support they can offer like getting medications -ideally I think the government gives us morphine and of late they started giving us a few drugs but we need more as compared to the need”.-KII 07-RHHJ |
|  |  |  | “Support the already existing hospices! We literally don’t have this big support from the government in terms of financial support -those who are already existing they support them”-KII 009-RHHJ |
|  |  |  | “Availability of the medicines-“In Busoga most facilities refer children here for morphine-sometimes they have the morphine at the facility but no one is willing to prescribe or even to dispense so there is a lot of capacity building that’s needed. There is also the issue of drug stock outs in the facilities”-KII 009-RHHJ |
